# Flow cytometry analysis of glucocorticoid receptor expression and binding in steroid-sensitive and steroid-resistant patients with systemic lupus erythematosus

**DOI:** 10.1186/ar2763

**Published:** 2009-07-14

**Authors:** Juan Du, Min Li, Denghai Zhang, Xiaoyan Zhu, Weiwei Zhang, Wei Gu, Yinglu Feng, Xiaofeng Zhai, Changquan Ling

**Affiliations:** 1 Department of Integrative Medicine, Changhai Hospital, The Second Military Medicine University, No.168 Changhai Road, Shanghai, PR China; 2 Department of Naval Medicine, The Second Military Medicine University; No.800 Xiangyin Road, Shanghai, PR China; 3 Department of physiology, The Second Military Medicine University; No.800 Xiangyin Road, Shanghai, PR China; 4 Department of Medical Technology, Changhai Hospital, The Second Military Medicine University, No.168 Changhai Road, Shanghai, PR China

## Abstract

**Introduction:**

Glucocorticoid (GC) therapy is the main treatment for systemic lupus erythematosus (SLE). However, some patients are resistant to these agents. Abnormalities of glucocorticoid receptor (GR) seem to be related to steroid resistance. This study evaluated GRs in T lymphocytes and monocytes of SLE patients by flow cytometry (FCM) using a monoclonal antibody (mAb) and FITC-Dex probes.

**Methods:**

Thirty-five patients with SLE before treatment and 27 age- and sex-matched normal controls were studied. Disease activity scores were determined before and after treatment and used to divide the patients into steroid-resistant (SR) and steroid-sensitive (SS) groups. GRs in T lymphocytes (CD3^+^) and monocytes (CD14^+^) were examined by FCM with GR-mAb and FITC-Dex probes before treatment. Peripheral blood mononuclear cells (PBMCs) were isolated for *in vitro *GCs sensitivity assays. The validity of FCM analysis of intracellular staining for GR with GR-mAb and FITC-Dex probes was evaluated through comparison with western blot and radioligand binding assay (RLBA) in U937 and K562 cells *in vitro*. One-way ANOVA, student's *t *test, linear regression and spearman correlation were performed.

**Results:**

A significant decrease in GR binding and the expression in K562 and U937 cells with 10^-6 ^M dexamethasone (Dex) was found compared with those without Dex. In addition, a positive correlation was found between FCM and RLBA as well as FCM and Western blot. The expression and binding of both CD3/GR and CD14/GR in SR patients with SLE, detected by FCM, were all lower than those in SS patients with SLE, whereas there was no significant difference in SS patients and controls. *In vitro *corticosteroid sensitivity assay indicated that PHA-stimulated tumour necrosis factor-α (TNF-α), IL-12 and interferon-γ (IFN-γ) secretion was significantly inhibited by 10^-6 ^M Dexamethasone in all controls and SS patients, compared with that in SR group, which confirms patient classification as SR and SS by disease activity index (SLEDAI) score.

**Conclusions:**

Abnormalities of expression and binding of the GR may be involved in tissue resistance to steroids in SLE patients. Determination of GR expression and binding by FCM may be useful in predicting the response to steroid treatment of SLE patients.

**Trial registration:**

Clinical trial registration number NCT00600652.

## Introduction

Glucocorticoids (GCs) are commonly used to treat autoimmune diseases such as nephrotic syndrome and systemic lupus erythematosus (SLE). However, there are so-called 'steroid-resistant' (SR) patients who fail to respond to treatment with GCs [1-3], the pathogenic mechanism of which is not fully understood. Glucocorticoid receptor (GR) seems to be related to the pathogenesis of steroid resistance, but the amount of GR in cells changes in different pathological states [4-9]. It was reported that lower GR binding affinity of peripheral blood mononuclear cells (PBMCs) correlated with a decreased responsiveness to treatment in patients with asthma as determined by a radioligand binding assay [4,5]. Also, in a cohort of 54 children with acute lymphoblastic leukaemia, lower expression of the GR detected by real-time PCR was associated with *in vitro *prednisolone resistance [6]. In contrast, two other studies suggested that the level of GR expression as assessed by western blot is not linked to *in vivo *or *in vitro *steroid response in children with acute lymphoblastic leukaemia [7,8].

The reasons for the aforementioned discrepancies between expression and binding capacity of GR and GC treatment sensitivity are complex, but one problem may lie in previous detection methods. Until now, the GR expression and binding have mainly been detected through western blot and radiolabelled receptor ligands (RLBA) in whole blood or PBMCs [4,5,7-11]. Such methods can not evaluate the GR expression or binding in individual cell types. Because GR expression and binding are different in respect to blood cell type, the variation in cell type percentages in different patients could prohibit reliable evaluation of the role of GR expression and binding with regard to hormone sensitivity [12]. It is important to discriminate between different cell types in evaluating response to steroid therapy.

Flow cytometry (FCM) is able to distinguish individual cells by size, cytoplasmic granularity and positive or negative expression of different receptors using fluorochromes conjugated to antibodies that recognise the proteins of interest [13,14]. Moreover with anti-GR monoclonal antibody (mAb) and fluorescein isothiocyanate (FITC) labelled dexamethasone (Dex) probes, FCM has the potential to detect the expression and binding of GR at the same time. It has been reported that high levels of GR expression do not always indicate a good response to treatment with GCs [15]. Similar observations have been made in studies of patients with ulcerative colitis [16-18]. The cause has not yet been explained. One possibility is that cytokines may play a role in steroid resistance by reducing the affinity of GR [19]. Therefore the detection of GR utilizing different modalities may be helpful to predict resistance to GCs therapy and to provide insight into potential mechanisms. To date, there have been only a few reports that attempt to correlate GR levels or binding with steroid response in patients with asthma, idiopathic nephritic syndrome and ulcerative colitis. However, there are few articles available in the literature concerning the significance of GR in assessing responsiveness to steroid therapy in SLE patients.

SLE is an autoimmune disorder that includes abnormalities in T lymphocytes, as well as hyperreactive B cells that produce autoantibodies. Recent studies supposed that monocytes, which produce IFN-α, might be involved in the pathogenesis of SLE [20]. Abnormal immunocytes contribute to the imbalance of cytokine homeostasis involved in immune dysregulation observed in SLE patients. Some studies have reported that IFN-γ, TNF-α, IL-6 and IL-12 were higher in SLE patients compared with normal controls [21-23]. Moreover, cytokines may play a significant role in steroid resistance [19]. *In vitro *Dex inhibition of Con-A-stimulated cytokine release has been used to examine the effect of corticosteroid in idiopathic nephritic syndrome [10]. GC therapy is essential for improving the pathologic state of patients with SLE. GRs are targets of GCs in moderating the immune disease. Therefore, the sensitivity of GRs in T lymphocytes and monocytes may account, in part, for the immunosuppressive efficacy of GCs. In the present study we evaluated the expression and binding of GR by FCM with GR-mAb and FITC-Dex probes in T lymphocytes (CD3^+^) and monocytes (CD14^+^) from SLE patients before GC therapy. In order to confirm patient classification as SR and steroid sensitive (SS) by SLE Disease Activity Index (SLEDAI) clinical score, the *in vitro *corticosteroid sensitivity assay was utilized.

## Materials and methods

### Patients and controls

Blood samples were obtained from 35 patients (4 males, 31 females, aged 20 to 63 years) with SLE in active phase who were diagnosed according to the American College of Rheumatology criteria [24]. Twenty-seven healthy volunteers (4 males, 23 females, aged 21 to 60 years) with no signs of acute or chronic illness and medicine intake, serving as controls. In EDTA-K_2 _tubes 5 ml anticoagulated blood was collected from the patients between 6:30 and 7:00 am when they were admitted to Changhai Hospital. The SLEDAI score [25] was determined in each SLE patient before and one month after steroid treatment (lower scores are an indication of improvement in disease activity). The patients with decreased SLEDAI score one month after steroid treatment served as the SS group and the remaining as the SR group [26]. The characteristics of the 35 patients with SLE are shown in Table 1. Patients 1 to 17 were included in the SR group and patients 18 to 35 comprised the SS group. We noted each patient's gender, years since diagnosis, symptoms, dosage of prednisolone and SLEDAI score. The mean ± standard deviation of age, gender ratio, years since diagnosis and dosage of prednisolone before study entry did not differ significantly between the SR and SS groups (Table 2). Normal controls and the two SLE groups are matched with respect to age and sex. Among the patients, 22 with relapsed disease (10 SR patients and 12 SS patients) took no other drugs in remission except for physiological doses of glucocorticoids (prednisone 5 mg/day orally). The expression and binding of GR as mean fluorescence intensity (MFI) of lymphocytes (CD3/GR) and monocytes (CD14/GR) containing receptors was determined as described by Marchetti and colleagues [27] in 200 μL fresh anticoagulated (heparinised) blood samples. Other blood samples were used for PBMC isolation. This prospective study was approved by the institutional review board for human research and informed consent was obtained from all subjects. The trial registration number is NCT00600652.

**Table 1:** Characteristics of 35 patients with systemic lupus erythematosus

Patient	Sex	Years since diagnosis	Symptoms	Dosage of prednisolone mg/day	SLEDAI	
					
					Before	After
SR group						
1	F	7	Abnormal serological data, fever	55	8	8
2	F	0	Abnormal serological data, fever, erythra, arthritis	50	12	12
3	F	0.25	Abnormal serological data, fever, proteinuia, defluvium	65	13	11
4	F	20	Abnormal serological data, erythra, pleurisy	40	7	7
5	F	0.25	Abnormal serological data, arthritis, defluvium	55	10	10
6	F	0	Abnormal serological data, erythra, defluvium, pyuria	60	14	14
7	F	0	Abnormal serological data, erythra, arthritis, pyuria	65	16	16
8	F	2	Abnormal serological data, erythra	55	8	8
9	M	3.8	Abnormal serological data, fever, erythra	50	9	9
10	F	1.3	Abnormal serological data, GN	65	12	12
11	F	0.42	Abnormal serological data, erythra, pyuria	60	10	10
12	F	4.08	Abnormal serological data, haematuria, proteinuia	60	12	11
13	F	14	Abnormal serological data, GN	60	10	10
14	F	0	Abnormal serological data, fever, erythra, pyuria	60	12	12
15	F	1.3	Abnormal serological data, fever, erythra, pyuria	60	13	13
16	F	0	Abnormal serological data, fever, defluvium, erythra	60	10	10
17	F	12	Abnormal serological data, fever, pleurisy, haematuria, proteinuia, pyuria	60	20	20
SS group						
18	F	0	Abnormal serological data, haematuria, proteinuia, pyuria, pleurisy	60	19	8
19	F	5	Abnormal serological data, haematuria, proteinuia, pyuria	60	18	10
20	F	0	Abnormal serological data, hematuria, proteinuia, CNS	70	22	10
21	F	0.08	Abnormal serological data, haematuria, proteinuria, red blood cell casts	60	18	13
22	F	0	Abnormal serological data, haematuria, proteinuria	60	14	8
23	F	25	Abnormal serological data, haematuria, proteinuria, Pe, erythra, pleurisy	60	20	10
24	F	5	Abnormal serological data, PHT	65	14	7
25	M	0	Abnormal serological data, fever, defluvium	55	9	5
26	M	0.08	Abnormal serological data, GN	65	12	9
27	F	3	Abnormal serological data, GN	60	10	2
28	F	0.08	Abnormal serological data, fever, erythra, defluvium	65	11	7
29	F	5	Abnormal serological data, fever, haematuria, proteinuia, pyuria	65	18	10
30	F	1	Abnormal serological data, fever, erythra	55	9	5
31	F	6.2	Abnormal serological data, fever, haematuria	60	11	5
32	M	0	Abnormal serological data, fever, proteinuia, defluvium	65	12	5
33	F	0	Abnormal serological data, fever, arthritis, proteinuia, pyuria	65	16	11
34	F	1.5	Abnormal serological data, fever, erythra, defluvium	65	11	5
35	F	3	Abnormal serological data, fever, Pe, arthritis, erythra	65	17	8

CNS = central nervous system; F = female; GN = glomerulonephritis; M = male; PHT = pulmonary artery hypertension; Pe = pericarditis; SLE = systemic lupus erythematosus; SLEDAI = SLE Disease Activity Index; SR = steroid-resistant; SS = steroid-sensitive.

**Table 2:** Comparison of characteristics among patients with SLE grouped according to steroid treatment response and normals*

Group	n	Gender (M/F)	Age, years	Years since diagnosis	Relapse	First treatment	Dosage of prednisolone mg/day
SR	17	1/16	34.4 ± 11.9	4 ± 6	10	7	57.6 ± 6.4
SS	18	3/15	39.7 ± 12.6	3 ± 6	12	6	62.1 ± 3.9
Normal	27	4/23	35.8 ± 13.3	-	-	-	-

*Values are the mean ± standard deviation. *P *> 0.5. Normal subjects and SLE patients are matched in their age and sex.

SLE = systemic lupus erythematosus; SR = steroid-resistant; SS = steroid-sensitive.

### GR-mAb-FCM analysis for the expression of GR

Phycoerythrin-CY5 (PE-CY5)-labelled antihuman CD3 and allophycocyanin (APC)-labelled CD14 mAbs (ebioscience, San Diego, CA, USA) were used for cell surface staining. Anti-GR mAb (abcam, Cambridge, UK; Mouse monoclonal (3D5) to Human Glucocorticoid Receptor, amino acid 150 to 175) and anti-mouse IgG-FITC (caltag, Burlingame, CA, USA) were used for the detection of GR. FITC-labelled mouse IgG1 isotype control (abcam, Cambridge, UK) was used for the control samples. A sample of 100 μL whole blood with an appropriate concentration of anti-CD3-PE-CY5 (10 μL) or anti-CD14-APC mAb (10 μL) or mouse IgG1 was incubated for 20 minutes at room temperature. Then the erythrocytes were lysed for 10 minutes with FACS lysing solution (Becton Dickinson, Franklin Lakes, NJ, USA). Cells were washed with PBS and fixed in 100 μL of 4% buffered paraformaldehyde (fixation buffer, ebioscience, San Diego, CA, USA) for 30 minutes at 4°C. Nonspecific binding site was blocked with 5% normal goat serum containing permeabilisation buffer for 30 minutes. The cells were then incubated with 100 μL permeabilisation buffer containing 4 × 10^-4 ^mg of anti-GR mAb or mouse IgG1 at 4°C for 60 minutes, washed twice in permeabilisation buffer, and then incubated with 100 μL permeabilisation buffer containing 2.5 × 10^-3 ^mg of goat anti-mouse IgG-FITC for 30 minutes. After extensive washing with permeabilisation buffer to remove unbound secondary antibodies, the cells pellets were resuspended in 300 μL fixation buffer.

### FITC-Dex-FCM analysis for the binding of GR

The protocol for surface staining and erythrocyte lysis were similar to analysis for the expression of GR above. Cell pellets were resuspended in 100 μL PBS containing 2 × 10^-8 ^M FITC-Dex (Molecular Probe^®^, Invitrogen, Carlsbad, CA, USA) for 60 minutes at 37°C in the dark with gentle mixing every 10 minutes. As controls, another tube was prepared adding a 500-fold excess amount of unlabelled Dex (sigma, Shanghai, China) 10 minutes before FITC-Dex. Finally, cells were washed twice and resuspended in 300 μL fixation buffer.

Cell samples were run on a FACSCalibur flow cytometer (Becton Dickinson, Franklin Lakes, NJ, USA) and analysed by CELLQuest software. At least 20,000 events in the light-scatter (SSC/FSC) lymphocyte and 8000 events in the light-scatter (SSC/FSC) monocyte region were acquired. CD3^+ ^and CD14^+ ^populations were identified and gated on PE-CY5 or APC plots. The relative quantity of GR (mean GR fluorescence) was expressed as MFI. The instrument calibration was performed daily by FACSComp software using CaliBRITE™3 beads.

Optimal dilution of probes used in FCM was obtained by comparing different dilutions. The majority of the unfixed cells were viable by propidium Iodide (PI) (50 μg/mL) staining (≥ 98%, data not shown). The specificity of staining in FCM was established by using non-specific mouse IgG1 and unlabelled Dex.

Additionally, to evaluate the reproducibility of FCM analysis of GR, GR expression and binding in T lymphocytes and monocytes from three normal young men were determined in three independent experiments. The blood samples were obtained at the same time of day on three different days. No significant differences were found among three experiments (data not shown).

### *In vitro *corticosteroid sensitivity assay

To perform the *in vitro *steroid sensitivity assay we measured the inhibitory effect of Dex on phytohaemagglutinin (PHA)-stimulated cytokines. PBMCs (2 × 10^6 ^cells per well), isolated from 4.8 mL anticoagulated blood of normal controls and SLE patients by Ficoll-Hypaque density-gradient centrifugation (Pharmacia, Piscataway, NJ, USA), were plated onto 96-well flat-bottomed plates (Corning, NY, USA) in triplicate and cultured at 37°C in the presence of 5% carbon dioxide. PHA at the dose of 10 μg/mL was used to stimulate the cells in the presence or absence of 10^-6 ^M of Dex. After 48 hours of culture, supernatants were collected and stored at -80°C for measurement of IL-12, TNF-α and IFN-γ levels by ELISA (ebioscience, San Diego, CA, USA). The minimum limits of detection for IFN-γ, IL-12 p40 and TNF-α were 8, 80 and 8 pg/mL, respectively.

Percent inhibition of cytokine secretion by steroid was calculated using the following formula [10]:



where x = cytokine secretion in Dex and PHA, n = cytokine secretion in RPMI alone, y = cytokine secretion in PHA alone. By using this calculation, the variations for cytokine secretion among and within individuals at different times were compensated.

### Comparion FCM analysis of GR with western blot and RLBA *in vitro*

Both human erythroleukaemia cell line K562 and monocytic/macrophage cell lineU937 were cultured in RPMI-1640 (GIBCO, Los Angeles, CA, USA) supplemented with 10% v/v FCS (PAA, Cölbe, Germany). To avoid interference from GCs compounds existing in FCS, cells were cultured in RPMI-1640 without phenol red (GIBCO, Los Angeles, CA, USA) two days before the experiment, which was supplemented with 10% v/v FCS-depleted of endogenous steroids (GIBCO, Los Angeles, CA, USA). Cells were seeded at a density of 300,000 cells per well in six-well flat-bottomed culture plates in triplicate and cultured in the presence or absence of 10^-6 ^M Dex after reaching confluence. After 48 hours of culture, cells were harvested by replacing the growth medium with RPMI-1640 without phenol red and collected by centrifugation at 500 g for five minute. Each cell pellet was then resuspended in 50 mL unsupplemented Joklik's media (250-fold dilution) at 25°C and allowed to sit for 10 to 20 minutes. This procedure was repeated, and finally each group was resuspended in 1.0 mL of unsupplemented Joklik's medium for cell count. This washing procedure removes most unlabelled Dex from the cells effectively. Intracellular staining for GR expression and binding was assessed by FCM as described above. At the same time, western blot and RLBA were performed as previously described [28,29].

### Statistics

One-way analysis of variance (ANOVA) was used to assess group differences in the GR expression and binding (mean ± standard error of the mean). For heterogeneity of variance, Kruskal-Wallis *H *test and Memenyi test were used. Age, gender, dosage of prednisolone and years since diagnosis were used as covariates. Student's *t *test and one-way ANOVA were used to assess cytokines measurements. Linear regression was used to assess correlation of results of GR detected by different methods. Spearman correlation was used to compare cytokine inhibition to the levels of GR or binding activity. SPSS version 15.0 (SPSS Inc, Chicago, IL, USA) was used for the analysis.

## Results

### Evaluation of FCM analysis of GR

In several tissues and cultured cells lines, administration of a GR agonist results in a significant down-regulation of GR. This result has been demonstrated *in vitro *by whole cell ligand-binding assays and western-blot in HeLa and COS-1 cells as well as cultured lymphocytes [30-32]. To evaluate FCM analysis of GR, we compared the result determined by FCM with that by RLBA and western blot using the model described above.

**Figure 1:**
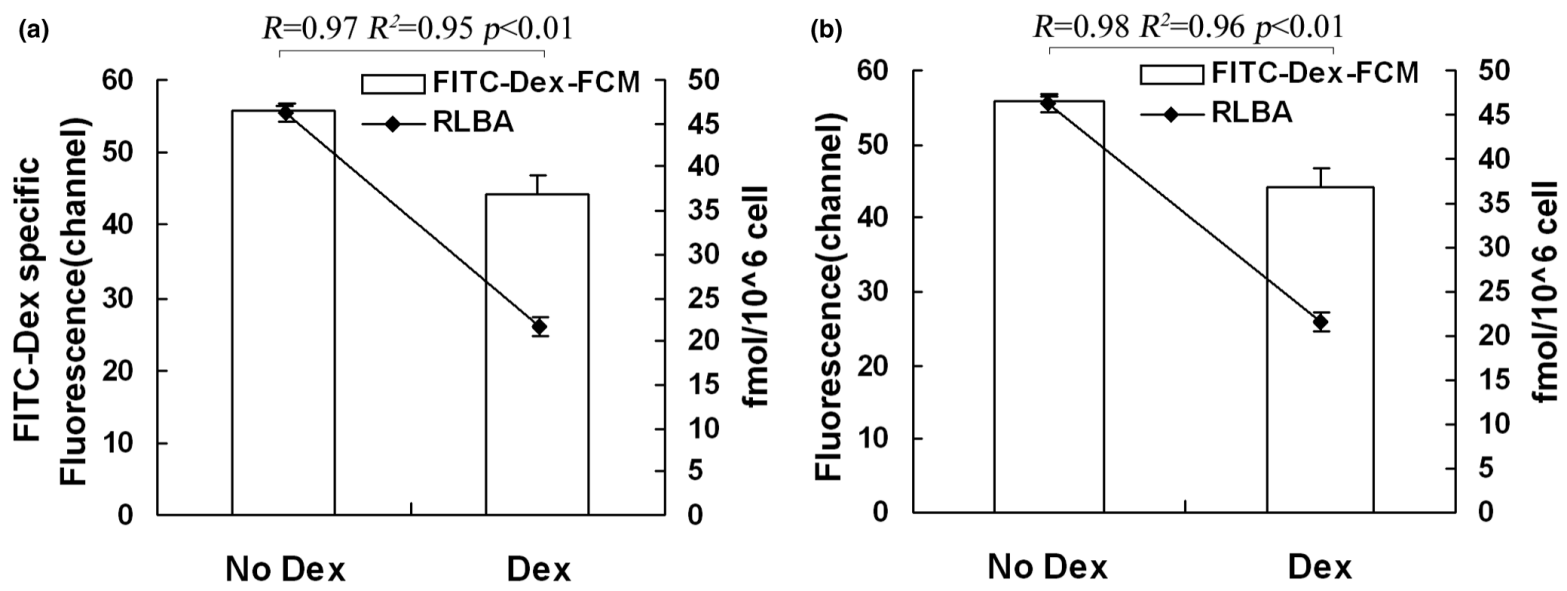
Evaluation of FCM analysis of GR binding by RLBA. Analysis of GR binding in **(a) **K562 and **(b) **U937 with and without 10^-6 ^M Dex by FITC-Dex-FCM and RLBA. Parallel FCM-FITC-Dex and radiometric assays were performed on the same day, using the same cell cultures to minimise variation. Specific FCM analysis was obtained as difference of mean channel number between total and nonspecific binding. By linear regression analysis, a positive correlation between results from the two methods was found. Dex = dexamethasone; FCM = flow cytometry; FITC = fluorescein isothiocyanate; GR = glucocorticoid receptor; RLBA = radiolabelled receptor ligand.

**Figure 2:**
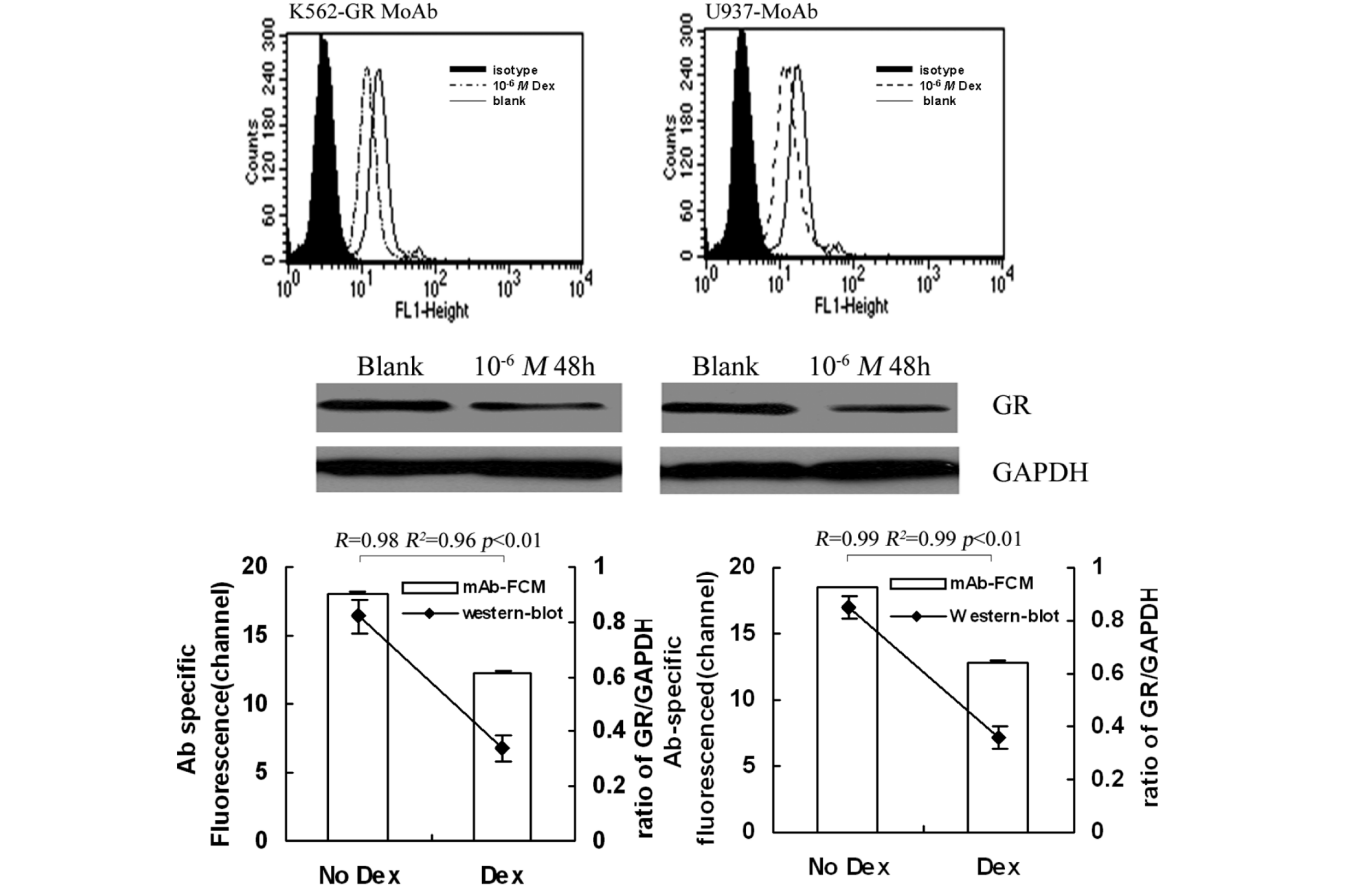
Evaluation of FCM analysis of GR expression by western blot. Analysis of GR repression in (left) K562 and (right) U937 with and without 10^-6 ^M Dex by GR-mAb FCM and western blot. Data are expressed as described in 2 figures at the bottom of Figure 2. Parallel GR-mAb FCM and western blot assays were performed on the same day, using the same cell cultures to minimise variation. Specific FCM analysis was obtained as difference of mean channel number between total and nonspecific binding. By linear regression analysis, a positive correlation between results from two methods was found. Dex = dexamethasone; FCM = flow cytometry; GR = glucocorticoid receptor; mAb = monoclonal antibody.

By FCM and RLBA, GR binding in K562 and U937 pretreated with 10^-6 ^M Dex decreased significantly (*P *< 0.01) compared with that without Dex, and a positive correlation was found between the two techniques (FITC-Dex FCM and RLBA, R^2 ^= 0.95, R^2 ^= 0.96, *P *< 0.01; Figure 1). Similarly, by GR-mAb-FCM and western blot, GR expression in K562 and U937 pretreated with Dex decreased significantly (*P *< 0.01) compared with that without Dex, and a positive correlation existed between the two techniques (R^2 ^= 0.96, R^2 ^= 0.99, *P *< 0.01; Figure 2).

### GR expression and binding of lymphocytes (CD3/GR) and monocytes (CD14/GR) in SLE patients and controls

**Figure 3:**
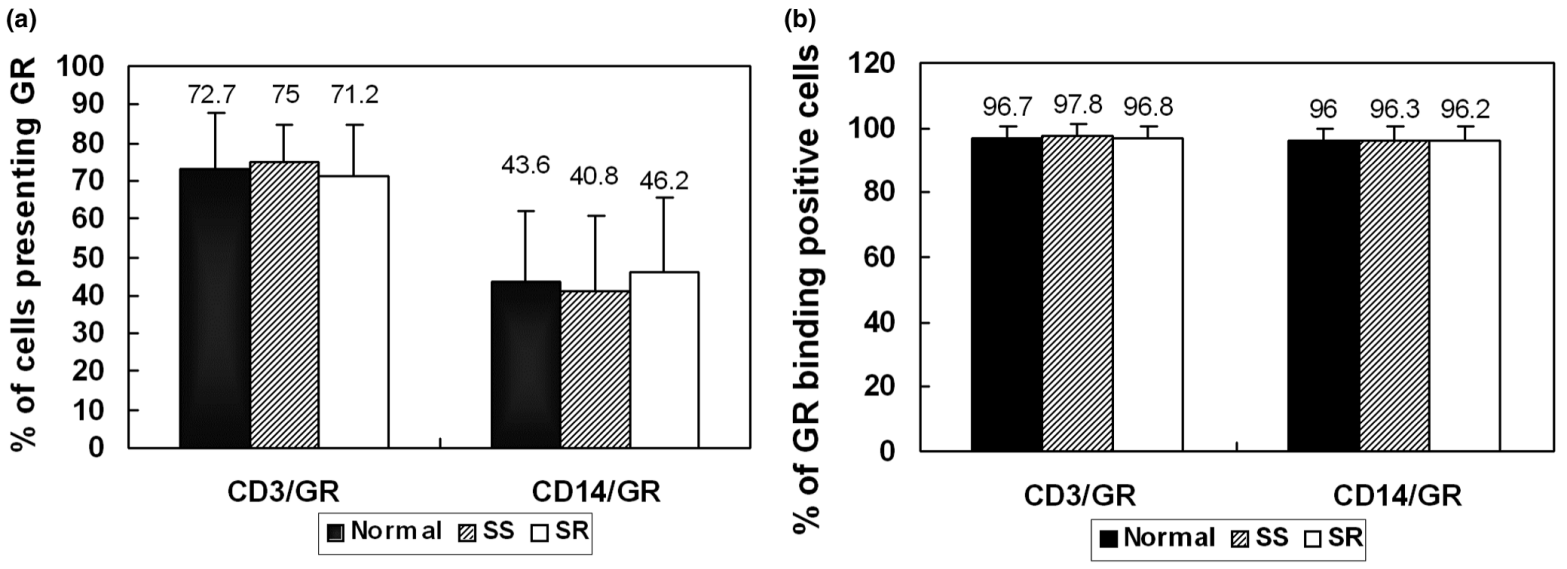
Percentage of GR-positive T lymphocytes and monocytes in SLE patients and controls. **(a) **Percentage of T lymphocytes and monocytes presentsing GR. The percentage of positive T lymphocytes and monocytes presenting GR in SR group did not differ from those in SS and the normal control groups (*P *> 0.05). **(b) **Percentage of GR-binding positive T lymphocytes and monocytes. The percentage of positive GR-binding cells was also similar among the SR, SS and normal control groups (*P *> 0.05). GR = glucocorticoid receptor; SLE = systemic lupus erythematosus; SR = steroid-resistant; SS = steroid-sensitive.

Disease activity scores were determined before and after steroid treatment (Table 1) and the SLE patients were divided into the SR and the SS groups accordingly. GR expression and binding in T lymphocytes (CD3/GR) and monocytes (CD14/GR) of SR, SS patients and controls were measured by FCM before treatment. The results showed that the percentage of GR-positive T lymphocytes and monocytes were 71.2 ± 13.4% and 46.2 ± 19.1%, respectively, in SR group, which did not differ significantly from the results of the SS group and the normal control group (*P *> 0.05, Figure 3a). The percentage of GR binding positive cells in detected cells was also similar among the SR, SS and normal control groups (*P *> 0.05; Figure 3b).

**Figure 4:**
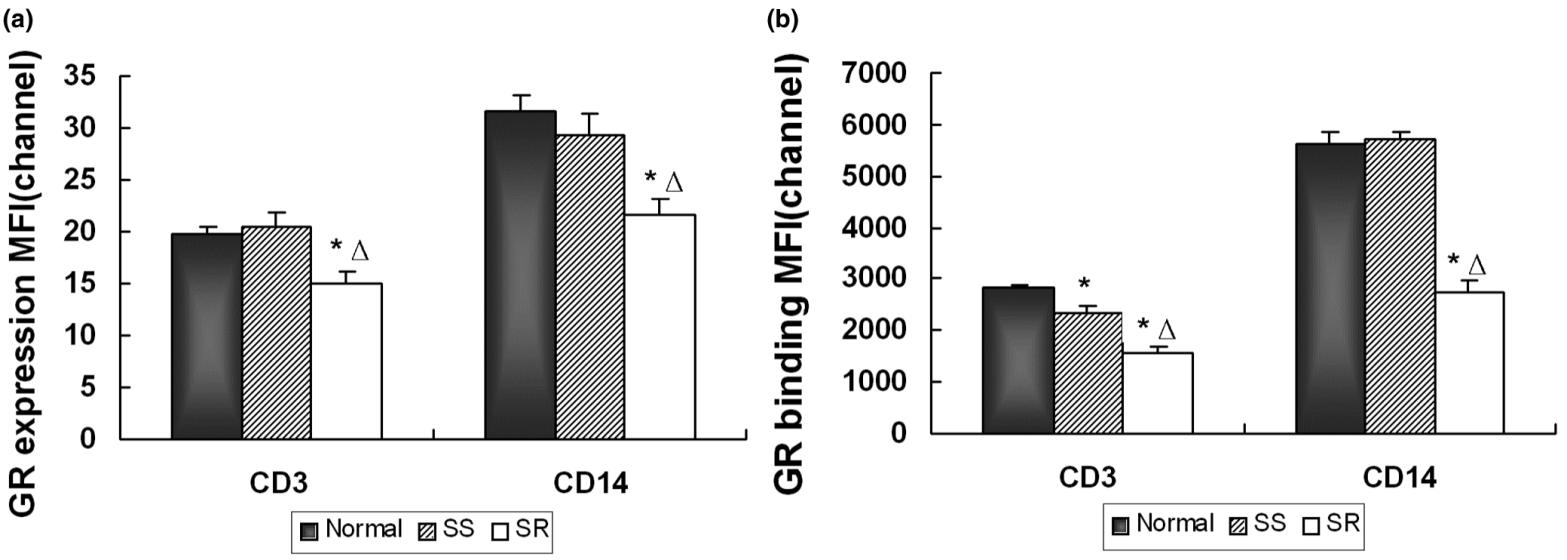
FCM analysis of GR in SLE patients and controls. Comparison of GR **(a) **expression and **(b) **binding in lymphocytes (CD3/GR) and monocytes (CD14/GR) among the SR group (n = 17) and the SS group (n = 18) with SLE, and the normal control group (n = 27). Bars show the mean ± standard error of the mean average fluorescence intensity of CD3/GR or CD14/GR detected by FCM before treatment. GR expression and binding in the SR group were significantly lower than in the other two groups. No differences were found between the SS group and normal control group in the expression and the binding of GR, except for the GR binding in CD3, which in the SS group was significantly lower than that in the control group. *P < 0.01, vs normal group, △ *P *< 0.01, vs SS group. FCM = flow cytometry; GR = glucocorticoid receptor; MFI = mean fluorescence intensity; SLE = systemic lupus erythematosus; SR = steroid-resistant; SS = steroid-sensitive.

However, GR expression in T lymphocytes (CD3/GR) and monocytes (CD14/GR) in the SR group were significantly lower than that in the SS and control group (*P *< 0.01; Figure 4a). There was no significant difference between GR expression in SS patients and that in normal controls (*P *> 0.05; Figure 4a). We also compared GR binding capacities among these three groups (Figure 4b). The results showed that GR binding in T lymphocytes (CD3^+^) in SR patients was significant lower than those in SS patients, which was lower than those in normal controls (*P *< 0.01). In monocytes (CD14^+^), GC binding in SR patients was significant lower than in SS patients (*P *< 0.01), which was no difference from those of normal controls (*P *> 0.05).

### Inhibition of cytokine secretion in SR and SS groups

**Figure 5:**
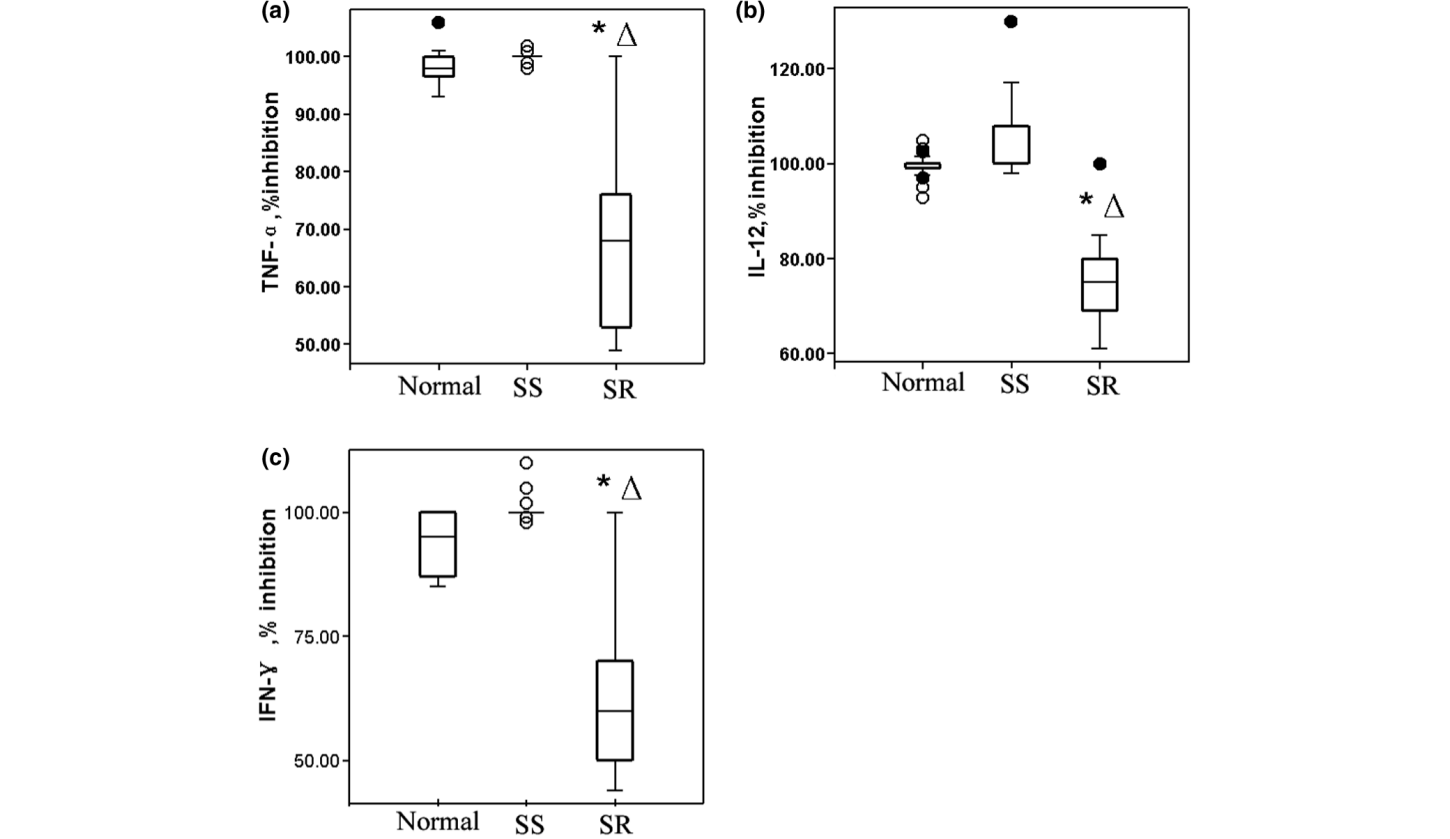
Inhibition of cytokine secretion in SR and SS groups. Percentage of inhibition of **(a) **TNF-α, **(b) **IL-12 and **(c) **IFN-γ cytokine secretion after PBMC incubation in RPMI with PHA plus 10^-6 ^M dexamethasone in normal controls, SS patients and SR patients. Calculation formula of percentage inhibition of cytokine secretion by steroid was described in Materials and Methods. *P < 0.01, vs normal group, △ *P *< 0.01, vs SS group. (solid circle is outliers, and hollow circle is extreme values). IFN = interferon; IL = interleukin; PBMC = peripheral blood mononuclear cells; SR = steroid-resistant; SS = steroid-sensitive; TNF = tumour necrosis factor.

We obtained PBMCs from 35 patients with SLE and normal controls before steroid therapy. Cytokine levels were measured in the supernatant collected from fresh cultured PBMCs in the basal condition and after stimulation with PHA alone or PHA plus 10^-6 ^M Dex. PHA-stimulated PBMCs increased TNF-α, IL-12 and IFN-γ secretion significantly compared with the basal production in the control (*P *< 0.01) and SLE groups (*P *< 0.01). The percentage inhibition of TNF-α, IL-12 and IFN-γ secretion after Dex in the controls, SS and SR patients are shown in Figure 5. With the 10^-6 ^M Dex treatment *in vitro*, TNF-α, IL-12 and IFN-γ secretion in the control and SS groups were significantly inhibited (*P *< 0.01). In contrast, the percentage inhibition of TNF-α, IL-12 and IFN-γ secretion were significantly lower in SR patients than those in SS and control groups (*P *< 0.01).

**Figure 6:**
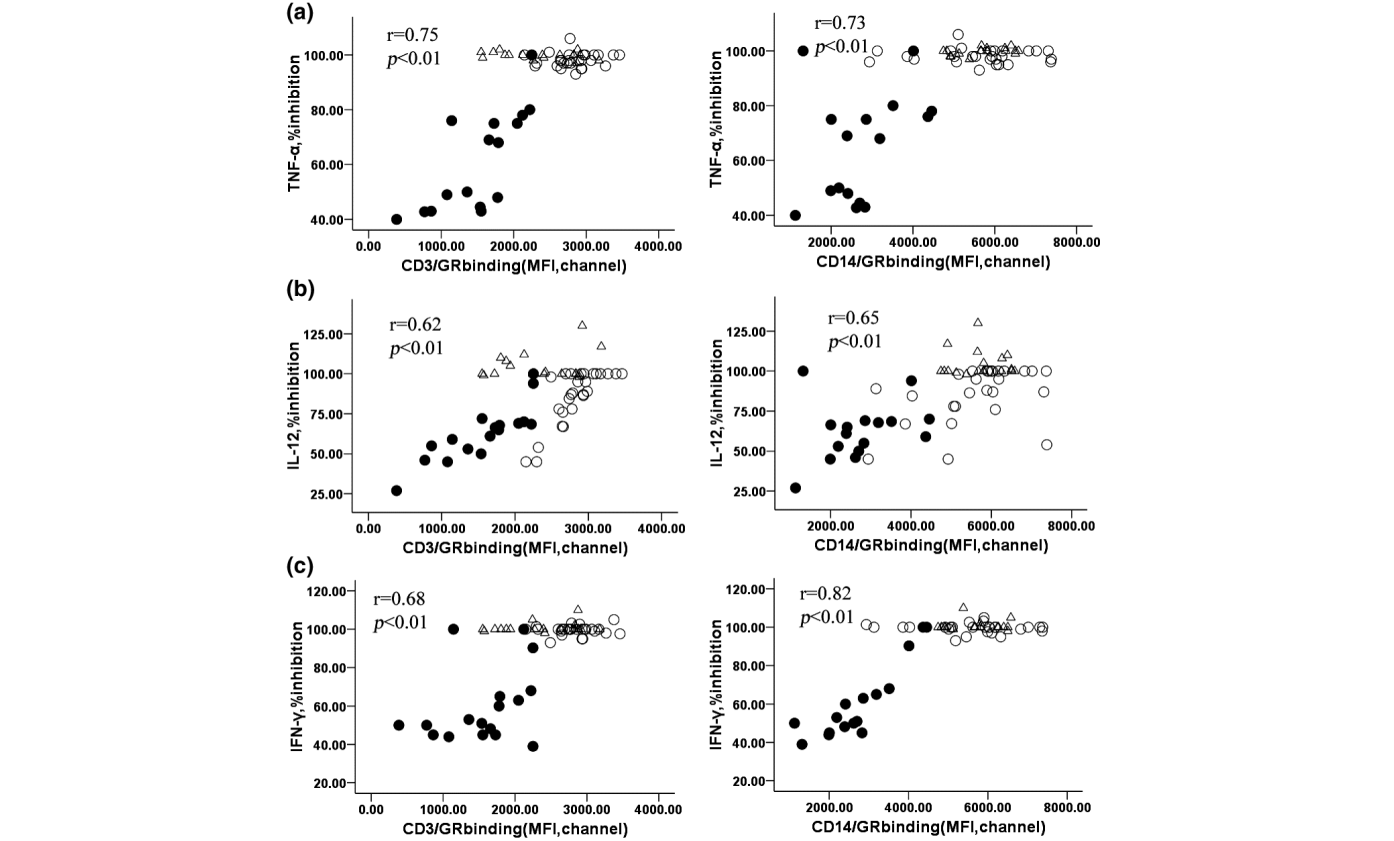
Correlation between GR binding and TNF-α, IL-12 and IFN-γ. Percentage inhibition of **(a) **TNF-α, **(b) **IL-12 and **(c) **IFN-γ cytokine secretion after PBMC incubation in RPMI with PHA plus 10^-6 ^M dexamethasone were correlated to GR binding in (left) CD3^+ ^and (right) CD14^+ ^subpopulation in normal controls, SS patients and SR patients. (hollow circle represents normal group, triangle represents SS group, solid circle represents SR group). GR = glucocorticoid receptor; IFN = interferon; IL = interleukin; PBMC = peripheral blood mononuclear cells; SR = steroid-resistant; SS = steroid-sensitive; TNF = tumour necrosis factor.

Moreover, the percentage inhibition of TNF-α, IL-12 and IFN-γ secretion in an *in vitro *corticosteroid assay were positively correlated with GR binding in T lymphocytes (CD3^+^) (r = 0.75, 0.62, 0.68, respectively) and monocytes (CD14^+^) (r = 0.73, 0.65, 0.82, respectively) of control, SS and SR groups (*P *< 0.01; Figure 6). Interestingly, there was no correlation between the inhibition of cytokine and GR expression (*P *> 0.05, data not shown).

## Discussion

It is known that GC therapy is the treatment of choice for patients with SLE. However some patients fail to respond to the treatment even when given high-dose GCs. For those patients, the treatment should be bolstered by synergising GCs with other immunosuppressants. If clinical response was predicted before therapy, synergised treatment might be performed at the beginning of the treatment to avoid side effects of chronic high-dose hormone therapy, which could improve the individual response to GC therapy and benefit more patients. GR seems to be related to the pathogenesis of steroid resistance. Thus, we hypothesised that detection of GR by a suitable method before treatment might be used to predict steroid response.

In the present study, we detected GR in PBMCs in SLE patients by FCM with GR-mAb and FITC-Dex probes, combined with cell surface staining for CD3 and CD14. Characteristics of patients, including age, male/female ratio, doses of prednisolone, disease history, etc, between SR and SS groups were coincident. Our results show that by FCM, the expression and binding of GR in SR patients with SLE were lower than those in both SS and control groups. However, there was no difference in the expression and binding of GRs in SS patients compared with those in controls except for the binding of GR in T lymphocytes. Similar results were also found in patients with nephrotic syndrome (data not shown). A previous study found decreases in the number and affinity of GR in SR patients with idiopathic nephritic syndrome and purported that the altered secretion of cytokines may be involved in tissue sensitivity to GCs [10]. Other studies have shown no change in GR of SR patients compared with those of SS patients and controls [7,8]. Different age distribution and pathological states may account for these conflicting results. In our study, the subjects were mainly adult patients with similar age distribution. We determined GR in CD3^+ ^and CD14^+ ^subset rather than the heterogeneous aggregate, which may play a role in these differences. Alternatively, GR was detected by FCM with different probes for the expression and binding at the same time. Moreover, the administration of GR agonists results in a significant down-regulation of the expression of GR *in vitro *and *in vivo*, so we detected GR of SLE patients in the active phase before the treatment rather than during the treatment. In summary, these data suggest that down-regulation of GR expression and binding might mediate steroid resistance in some patients with SLE.

We also evaluated the percentage of positive lymphocytes and monocytes presenting GR in SLE patients and normal controls. No difference was found between the results of patients and controls. Similar results were obtained by Wasilewska and colleagues [33]. From these results, we infer that the expression and binding of GR on a per cell basis, but not the overall positive rate of GR, might be correlated with steroid response.

In addition, we performed an *in vitro *corticosteroid sensitivity assay in patients and normal controls. *In vitro *Dex inhibition of Con-A stimulated cytokine release has been used to examine the effect of corticosteroids in idiopathic nephritic syndrome [10]. In our study, the control and SS groups presented higher inhibition of cytokine secretion than SR group, which confirms patient classification as SR and SS by SLEDAI score. As a result, the detection of GR by FCM before steroid therapy as well as *in vitro *corticosteroid sensitivity assay may predict clinical response to the treatment more accurately. As to the relation of GR and inhibition of cytokine secretion, there was a positive relation between the GR binding in two subsets (T lymphocytes and monocytes) and the inhibition rate of cytokine secretion, which was different from the GR expression. However, whether the low binding capacity of GR is contributing to the reduced inhibition rate of cytokines or the systemic inflammation induced GC resistance [34,35] need to be further discussed.

GR binding and expression have been detected recently by RLBA and western blot, separately. However, the two methods could not evaluate individual cells or specific cell types in a given tissue sample. Moreover, it is challenging to use the two methods in a clinic for they are time consuming, expensive and radioactive materials are unavoidably involved.

FCM is a convenient tool for determining protein expression of cell surface receptors and has also been shown to be useful in identifying expression of intracellular proteins in permeabilised cells [36]. Especially when complex mixtures of cells are present, FCM can be used to sort subpopulations of cells and therefore identify GR expressed in specific cell types. In a recent study, a method for the quantification of GR by FCM was developed in different cell lines [37]. Although it established the method which could detect GR, even quantifying the number of receptor molecules per cell, it seems that they did not reflect GR from various points of view. A few studies in the literature indicated that a high level of GR did not always relate to a satisfying response to GC therapy [38]. Similar observations have been made in studies of patients with ulcerative colitis [39-41]. As a result, the analysis of GR expression and binding was important to the evaluation of the response to GC therapy.

Detection of GR expression and binding by FCM using mAb and fluorescein ligand probes at the same time was first reported in clones of CCRF-CEM human leukaemic cells [27]. In the current study, it was first used to identify biomarkers that clinically predict steroid response. To confirm the GR measurements by FCM, RLBA, which had been used for GR binding detection, and western blot, which has been used for GR expression detection, were performed at the same time and the results were consistent between FCM and western blot or BLRA.

## Conclusions

Our data showed that the GR expression and binding in SLE patients was lower in the SR group than that in the SS and control groups. FCM was a reliable and reproducible method for measuring GR in experimental and clinical settings. The clinical response to steroid therapy of patients with SLE may be predicted using FCM analysis of GR. Further studies are needed to expand sample size and determine the normal scope of GRs.

## Abbreviations

ANOVA: analysis of variance; APC: allophycocyanin; Dex: dexamethasone; ELISA: enzyme-linked immunosorbent assay; FCM: flow cytometry; FCS: fetal calf serum; FITC: fluorescein isothiocyanate; GC: glucocorticoid; GR: glucocorticoid receptor; IFN: interferon; IL: interleukin; mAb: monoclonal antibody; MFI: mean fluorescence intensity; PBMC: peripheral blood mononuclear cells; PBS: phosphate-buffered saline; PCR: polymerase chain reaction; PE-CY5: phycoerythrin-CY5; PHA: phytohaemagglutinin; PI: propidium Iodide; RLBA: radiolabelled receptor ligand; SLE: systemic lupus erythematosus; SLEDAI: SLE Disease Activity Index; SR: steroid-resistant; SS: steroid-sensitive; TNF: tumour necrosis factor.

## Competing interests

The authors declare that they have no competing interests.

## Authors' contributions

CL conceived the study, participated in its design and coordination. JD and ML contributed equally in study design, collecting samples from patients, FCM analysis of GR, western blot and drafting the manuscript. DZ participated in study design, performed most of the statistical analyses and revised the manuscript. XZ participated in study design, interpretation of data and revision of the manuscript. WZ contributed in FCM analysis of GR. WG participated in RLBA and interpretation of statistical analyses. YF and XZ contributed with samples from controls and participated in revision of the manuscript. All authors read and approved the final manuscript.
